# Food taboos: their origins and purposes

**DOI:** 10.1186/1746-4269-5-18

**Published:** 2009-06-29

**Authors:** Victor Benno Meyer-Rochow

**Affiliations:** 1 School of Engineering and Sciences, Jacobs University, D-28725 Bremen, Germany; 2 Department of Biology, University of Oulu, SF-90014 Oulu, Finland

## Abstract

Food taboos are known from virtually all human societies. Most religions declare certain food items fit and others unfit for human consumption. Dietary rules and regulations may govern particular phases of the human life cycle and may be associated with special events such as menstrual period, pregnancy, childbirth, lactation, and – in traditional societies – preparation for the hunt, battle, wedding, funeral, etc. On a comparative basis many food taboos seem to make no sense at all, as to what may be declared unfit by one group may be perfectly acceptable to another. On the other hand, food taboos have a long history and one ought to expect a sound explanation for the existence (and persistence) of certain dietary customs in a given culture. Yet, this is a highly debated view and no single theory may explain why people employ special food taboos. This paper wants to revive interest in food taboo research and attempts a functionalist's explanation. However, to illustrate some of the complexity of possible reasons for food taboo five examples have been chosen, namely traditional food taboos in orthodox Jewish and Hindu societies as well as reports on aspects of dietary restrictions in communities with traditional lifestyles of Malaysia, Papua New Guinea, and Nigeria. An ecological or medical background is apparent for many, including some that are seen as religious or spiritual in origin. On the one hand food taboos can help utilizing a resource more efficiently; on the other food taboos can lead to the protection of a resource. Food taboos, whether scientifically correct or not, are often meant to protect the human individual and the observation, for example, that certain allergies and depression are associated with each other could have led to declaring food items taboo that were identified as causal agents for the allergies. Moreover, any food taboo, acknowledged by a particular group of people as part of its ways, aids in the cohesion of this group, helps that particular group maintain its identity in the face of others, and therefore creates a feeling of "belonging".

## Background

Years ago a student asked me the following question: "Why don't all animals eat the same kinds of food?" This may have sounded a stupid question, but it is not as trivial an enquiry as one might have thought initially. Afterall, to grow and survive, animals all need the same basic things: carbohydrates, protein, fats, some minerals and water." So, why do we have this diversity of food specialists on Earth? Why are there herbivores, carnivores, detritovores, insectivores, fungivores, coprophages, xylophages and many more?

Although it is true that all heterotrophic organisms need the same fundamental food stuffs, it is easy to understand that on account of their different sizes, different anatomies, and different habitats, different species must make use of different food sources to satisfy their needs. A cat would happily devour the meat of an antelope and a lion would not reject a mouse, but both are not built for these kinds of food items. A tree-dwelling leaf-eater does not graze on the ground and a grazer does not climb trees. Pond snails may love lettuce, but they can never leave their watery realm. Moreover, it is a "Law of Nature" that, where there is an underexploited resource, it usually does not take long before such a resource is 'discovered' and used by some organism. Yet, intense competition for one and the same kind of food by two species ultimately would lead to the extinction of one of them or it would result in the two species occupying different niches, either in connection with the food itself or the timing of feeding [1,2].

It is, thus, easy to understand why different *species *of animals with different anatomies and habitat preferences should use different food items, but food specialists *within *a species also occur and it is then less obvious why individuals of one and the same species should exploit different resources. It becomes really tricky, when some adults of the same gender, species, and overall physical built nevertheless vary in relation to their food preferences. Intraspecific competition may be involved, differences in hunting and/or collecting skills and strategies, acquired through learning or chance discovery, could be the reason, and there could even be an outwardly not visible physiological basis for such kinds of behaviour. Yet, no ecologist or zoologist would use the term "food taboo" to describe intraspecific food preferences of this kind in animals, but in connection with humans we do use the term "food taboo". We use it (or refer to "prohibitions") to distinguish the deliberate avoidance of a food item for reasons other than simple dislike from food preferences. In non-human mammals, dominant individuals may force weaker ones to accept less sought-after food items, and a possible liking for these originally reluctantly accepted food items may in turn develop [2,3]. Some aspect of this scenario may also apply to human societies, because food taboos can be imposed on individuals by outsiders, or by members of the kinship group to manifest themselves through instruction and example during upbringing [4].

Probably food taboos (as unwritten social rules) exist in one form or another in every society on Earth, for it is a fact that perhaps nowhere in the world, a people, a tribe, or an ethnic group, makes use of the full potential of edible items in its surroundings [5-10]. One of many examples, although an especially well-studied one, involves the Ache people, i.e., hunters and gatherers of the Paraguayan jungle. According to Hill and Hurtado [6], the tropical forests of the Ache habitat abound with several hundreds of edible mammalian, avian, reptilian, amphibian and piscine species, yet the Ache exploit only 50 of them. Turning to the plants, fruits, and insects the situation is no different, because only 40 of them are exploited. Ninety eight percent of the calories in the diet of the Ache are supplied by only seventeen different food sources.

Although mere avoidance of potential food (for whatever reason) does not in itself signify a food taboo, it is easy to see how regular avoidance can turn into a tradition and eventually end up as a food taboo [7,8,10]. But what is it that leads to the regular avoidance? Social anthropological research on eating and food taboos (cf., reviews [7-11]) has frequently invoked utilitarian [7-9] and magico-religious motives [10] or seen the dichotomy between positive and negative rites as a basis for food taboos [11,12]. A functionalist's explanation of food taboos as mechanisms for conserving resources as well as a person's health, have been less popular (cf., [13]), although there is good evidence in support of both [14-19]. Yet even rituals and taboos based on spiritual, religious, and magic ideation must have had a "history" and somehow 'got going' [7-11,20-23]. Therefore, given that food taboos can involve plants as well as animals, solids as well as liquids, hot as well as cold categories, wet and dry items, etc. [7-9,12-15], this review, rather than attempting to provide a complete list of food taboos operating in human societies, will instead present examples of food taboos in selected human groups that illustrate some of the wide spectrum of food taboo origins. The five examples chosen reflect the author's own cultural background (Jewish dietary laws), or are based on original field research by the author in Central Australia, Papua New Guinea, and India, or refer to other persons' published work (e.g., food taboos of the Orang Asli by [24]).

## Methods

Based on the authors own experience, observations, recordings, and interactions with locals, examples of Jewish dietary laws and Hindu practices form the basis of examples 4 and 5. Research stays in India of 2 months (Meghalaya and Nagaland) and three weeks (Karnataka and Goa) during sabbaticals in 1990 and 2005 as well as a Brahmin Indian wife further helped gathering the necessary information for the section on Hindu food taboos.

Field work by the author in Papua Niugini of several weeks each in 1972 (Onabasulu and neighbouring tribes), 1998, 2002, and 2004 (Kiriwina), during which the author stayed with the locals in their villages or homesteads and then studied the locals' entomophagic practices as well as food taboos, forms the basis for the information given in example 2. Information in the field was always gathered from more than one informant (although it has to be mentioned that the informants were all males). Examples 1 and 3 (Orang Asli and Mid-West Nigerian food taboos) were chosen from the literature available, because they illustrated yet other aspects and reasons for food taboos, not covered in the earlier mentioned examples. Thus, the selection of the examples represents a mixture between emic experiences from within a culture and etic approaches, i.e., results of field work amongst cultures other than the author's own and research carried out by additional investigators on yet further cultural entities. The reason for the selection of the examples was twofold: to demonstrate the existence of very different possible food taboo reasons and to re-ignite interest in this important field of inter-disciplinary research.

## Results

### Example 1: The Orang Asli food taboos

The term 'Orang Asli' describes a variety of aboriginal tribes, nowadays confined to the forests and forest fringes of West Malaysia. Food taboos amongst these people have been recorded by Bolton [24]. In the context of this review, the Orang Asli were chosen as an example of a people, in which food taboos appear to serve a double-purpose: the spiritual well-being of individuals and resource partitioning.

Human flesh is never eaten and animals, which the Orang Asli have kept as pets or have reared, are also protected. They can be sold, though, or given away to others, who then would have no qualms of consuming them. An animal that is capable of feeding on a human being will not be eaten as it conceivably could contain some "humanness" in it.

Small lizards and leeches are considered to be unclean to the jungle Orang Asli. Should a leech, for example, accidentally drop into the cooking pot, all its contents will be regarded as contaminated and thrown away. Poisonous and harmful animals are also taboo, but the dangers that result from eating certain species are frequently less real or physiological than spiritual/psychological. Thus, the crow is thought to be poisonous and is rarely eaten. Likewise, any small, crawling animal living in or on the soil, is usually left alone for fear it might be dangerous.

Since all animals are considered to possess spirits, many Orang Asli will start their weaned children of more than 4 years of age on small animals: fish, frogs, toads, small birds and water snails. When the child gets a bit older, rats and mice can be added to the list of edible species.

At 20 years of age the human spirit is deemed to be strong enough to successfully compete with the spirits of small monkeys, bat species, cats, anteaters, deer, turtle, larger birds, and even the Malayan bear. Later in age snakes, gibbons, and bigger animals, including the elephant, no longer remain taboo.

Pregnant women have strict food taboos to observe and must restrict themselves to rats, squirrels, frogs, toads, smaller birds and fishes, that is animals which are small and thought to possess "weak" spirits. Moreover, rodents may be eaten only if caught by the pregnant woman's husband or a near relative and she must eat the whole rodent by herself. Fish must also be caught by a near relative (but never with a spear or with the help of explosives).

After childbirth, the mother normally eats gruel for a week and for 6 weeks thereafter has to eat on her own. She continues to observe food taboos, but her husband, who observed the same food restrictions as his pregnant wife, is then no longer bound by them. Special 6-day food taboos may be "prescribed" by a medicine man for any sick person that seeks his advice.

Although the food taboos of the Orang Asli are not totally absolute, men are always ready to remind the younger women and children of the dangers of breaking them and of eating meat of new and unfamiliar species.

### Example 2: Food taboos of Papua New Guinea tribals

In Papua New Guinea ('Niugini" in Pidgin-English) with her multitude of peoples and cultures, food taboos are particularly varied. The example chosen illustrate that many food taboos are designed to protect humans from health hazards real and assumed. Yet, a tendency by some section of the society to safeguard exclusive rights to certain food items is also obvious.

Onabasulu and neighbouring tribes with institutionalized homosexuality, like the Kaluli and others, regard with great suspicion any organism that lives or burrows in the soil [25]. Even harmless earthworms are detested. Illnesses are thought to frequently stem from the wrong food intake: stomach ache sufferers must avoid juicy fruits, such as watermelons, pawpaw, cabbage and the introduced pineapple.

Women are thought to be permanently in this 'sickly' and 'runny' state, because of recurring menstruations and are not allowed fresh meat, juicy bananas and all fruits of the forest of red colour. If a menstruating woman eats a fresh animal caught in a trap, it is thought that future traps will not fall; if the animal was caught with a dog, it is feared that the dog will lose its ability to find scent. Similarly, bananas and pandanus: if a menstruating woman happens to eat some of these fruits, it is believed that the trees will then cease to bear. A woman herself must leave the communal longhouse and move to a shack some distance away for the duration of her period. If she should cook or step over food, those who eat it, particularly her husband, will become "ill with cough and possibly die" [26]. Mature women must not eat fish and when pregnant are not even permitted eggs. Young unmarried men receive the best food and have to obey the smallest number of food taboos. When married, they, like their wives, can no longer eat fresh, but only smoked meat.

In the Kiriwina (Trobriand) Islanders, pregnant women, too, have a considerable amount of food taboos to observe: fishes that lead a cryptic life or like to attach themselves to corals are not to be eaten by a pregnant woman, because this might cause her to have a complicated birth. Similar beliefs are attached to bananas, pawpaws, mango, and other fruits; they are thought to either cause a hydrocephalus, club-foot, distorted belly or give rise to other deformities in the newborn [27,28].

In addition to these food taboos, different ones, affecting men, also exist. If the men intend to go fishing for sharks, they not only have to abstain from sexual intercourse for a while, but they also have to fast (*posuma*) and drink a large quantity of saltwater beforehand. Flatfish, including soles and stingrays, as well as a considerable number of other species of fish are taboo, and during the turtle season no garden work is to be carried out.

Food unfit for human consumption in one village because of taboos, may, however, be traded for the permitted item from others, who observe other taboos. For example, the socially excluded inhabitants of the village of Boitalu are the only people on the Kiriwina Islands that can eat wild pig and wallaby (a small species of kangaroo).

Particularly strict taboos govern what chiefs are permitted to eat. In the northern part of Kiriwina they may eat only fried or roasted things, stewed and boiled food being banned. In the south, however, the village chiefs are the only ones allowed to violate against the flatfish and stingray taboo.

### Example 3: Food taboos in Mid-West Nigeria

The continent of Africa, because of its size, presents an enormous variety of food taboos. In many parts fresh milk is avoided by adults, although for the Masai, Fulbe, Nuba and other East African groups this commodity is thought to represent a particularly wholesome food for young men and warriors [29]. Observations on food taboos of the inhabitants of mid-west Nigeria were chosen as they represent a particularly good example of a people, in which food taboos appear to have been imposed on society mainly to serve the interests of the 'strongest' section, i.e., the reification of social hegemonies of the society: in particular the menfolk [30].

In the mid-west state of Nigeria, meat and eggs are not usually given to children, because parents believe it will make the children steal [30]. Gizzards and thighs of ducks are eaten by the elderly; children can only have the lower legs or sometimes the head. Frequently coconut milk and liver is taboo for children, because it is believed that "the milk renders them unintelligent, whereas the liver causes abscesses in their lungs" [30].

In some parts of Ishan, Afemai, and Isoko Divisions pregnant women avoid snails, whereas pregnant women of the Asaba Division are neither allowed to eat eggs nor drink milk, "because it is feared the children may develop bad habits after birth" [30]. Woen tribals of the Ika Division are forbidden to consume porcupine as that is thought to cause a delay in labour. Interestingly, the opposite (an easy delivery) is expected from some pregnant Urhobo women, who have consumed food leftovers from a rat. Following delivery, young mothers in parts of Benin and Ishan Divisions must not consume oil or fresh meat and in parts of Ishan, palmnut soup is forbidden for 30 days postpartum.

Men have fewer food taboos to observe, but nevertheless some also exist. Snail consumption may weaken a warrior's strength and to kill and eat some legendary animals that have helped a particular tribe in the past during intertribal warfare is totally forbidden. Thus, in some areas the partridge or bush fowl is not eaten; in others it is some water reptile or the porcupine or even the sheep that are protected by the food taboo. Beans are one of the plant species that are not eaten, because they are believed to cause stomach disorders.

### Example 4: The Hindu food taboos

The Hindu food taboos were chosen as example nr. 4 to illustrate how, in this case, the spiritual aspect dominates all food taboos. The concept of re-incarnation and the sanctity of life lies at the root of these food taboos, but resource conservation and safe-guarding health play a role as well.

In the Vedic Hindu Society there is a subdivision into 4 castes on the basis of labour: Brahmin (priestly), Kshatriya (defence), Vaisya (agriculture and business), and Shudra (menial labour). Lord Krishna compared the community to a human body, in which the Brahmin caste represents the head, and the others the arms, legs and bowels. Brahmins never handle any meat, fish, or eggs let alone eat any of these foods. A Brahmin cannot even imagine bringing such foods into the house. Furthermore, many orthodox Brahmins abstain from cooking or eating onion and garlic as they are said to increase passions like anger and sex drive. Milk and milk products are consumed, but said to be very sacred as the cow is held in the highest regard as "a holy mother".

Although the people belonging to the three other castes sometimes partake in fish, eggs, and even meats (normally only chicken, goat, or mutton), these are never to be cooked or eaten during religious occasions, marriages, times of mourning, breaking religious fasts, pilgrimages, and similar times. Certain special religious festival days (as well as Mahatma Gandhi's Day) are declared by the Indian Government as "Meatless Days" when no meat is sold anywhere. In castes, in which meat-eating does occur, widows are tabooed from eating meat, fish, or eggs so as to keep their passions low. On the l1th day after New Moon and Full Moon (Ekadasi) many Hindus abstain from eating grain, which otherwise is their staple food. Pregnant women are restricted from eating pawpaw and jackfruit as substances in these fruits are feared to have abortive influences.

During any religious ceremony (and for a Brahmin, every day is governed by strict religious schedules) the offering of food to the gods always precedes food intake. Food, thus, becomes sanctified and is called 'Prasad' (i.e., God's Mercy), which is then partaken. This practice follows from the ancient scripture "Bhagavad Gita" [31], in which the Lord says: "If one offers Me with love and devotion a leaf, a flower, fruit, or water, I will accept it" (Text 26) and "...all that you do, all that you eat, all that you offer and give away as well as austerities that you may perform, should be done as an offering unto Me" (Text 27). "In this way you will be freed from all reactions to good and evil deeds and by this principle of renunciation you will be liberated and come to Me" [31].

Hindus do believe that plants also have life, though in a more sedate and sedentary form. The use of plants as food is considered less sinful than taking the lives of animals, but they must not be broken or harvested after dark. The saying "You are what you eat" is explicitly mentioned in the Bhagavad Gita (Chapter 17: [31]): "Foods in the mode of goodness increase the duration of life, purify one's existence and give strength, health, happiness and satisfaction. Such nourishing foods are sweet, juicy, fattening, and palatable. Foods that are too bitter, too sour, salty and pungent, dry and hot, are liked by people in the modes of passion. Such foods cause pain, distress, and disease. Food cooked more than three hours before being eaten, which is tasteless, stale, putrid, decomposed and unclean, is food liked by people in the mode of ignorance". Thus, although this powerful message does not contain precise instructions to "do" or "not to do", it describes the effects of different kinds of food and leaves the final choice to the individual. The non-selected foods may therefore be declared food taboos by society.

The Situation with regard to liquids is fairly similar. Intoxicants are plainly said to put a person's mind off the natural course and, hence, puts the person into more passion and ignorance. Alcohol and narcotics are, therefore, forbidden and will not enter the household of a traditional Hindu family.

### Example 5: The Jewish dietary laws

Jewish dietary laws, containing some of the sentiments found also in the Hindu food taboos, have been chosen to illustrate how food taboos with origins steeped in religion, promotion of health, and protection of life combine to create a set of rules that foremost and for all unite a people and create group-cohesion.

On the day of the Atonement (Yom Kippur) no Jew will eat or drink anything for 24 hours (and on the ninth of the month of 'Av' many will fast again). During the first nine days of the month of 'Av', as an expression of mourning, no meat whatsoever is eaten. On Pessah (Passover) nothing that is leavened (in other words ordinary bread) is consumed or enters a Jewish home.

Certain kinds of food have become associated with particular seasons or festivals: the matzah has become the bread of affliction on Pessah; 'gefillte fish' is a common dish on the Shabbat eve; a Rosh Hashanah (the Jewish New Year) without apples and honey is impossible to imagine, and hamantaschen and kreplach are foods symbolical of the feast of Purim [32]. Yet, all through the year a Jew is conscious of his/her Jewishness through complex dietary laws, collectively termed 'kashrut'. Milk or milk-products (i.e., 'Milchiges' in Yiddish) must never be consumed together with meat (i.e., 'Fleischiges' in Yiddish). Plates, pots, cutlery, and other utensils used in connection with meat-containing foods must be kept separate at all times from those used with other foods.

To be classified as permitted (i.e., kosher), an animal must both chew the cud and have a cloven hoof, birds have to have wings, and aquatic organisms must possess both fins and scales [33]. Shrimps, oysters, lobsters, creatures that creep on the ground, reptiles and worms found in fruits or vegetables are all prohibited. To ingest blood of any animal is strictly forbidden, and to be fit for consumption "beast and fowl must be slaughtered according to the law and if they are not of a domesticated species their blood must be covered with earth after slaughter" [34].

An animal that has died naturally is considered unfit for consumption as is a torn or mauled animal. Also prohibited is the sinew of the thigh (*gid hanasheh*) of any animal. The only permitted way of slaughter is with "an exceedingly sharp knife without the slightest notch so as to make the taking of a life as painless a procedure as possible" [35]. Slaughtering an animal and its young on the same day is prohibited and there is also a requirement to release a parent bird before taking the chicks [36]. As one of the seven Noachidic Laws, the prohibition to eat flesh of a living animal applies to Jew and non-Jew alike. The rabbinical attitude towards hunting animals for pleasure is entirely negative [35].

Interpreting the biblical record, mankind was not allowed to eat any meat at all until after "the Flood", although as part of the holy sacrifice of animals to God the consumption of kosher meat had been allowed [37,38]. Later, when the entire Jewish people became considered a "kingdom of priests", the priestly rules in relation to the consumption of "clean" (kosher) meat were extended to the whole community. Even then, only special persons can actually take an animal's life. It has to be a 'Shohet', the Jewish ritual slaughterer, whose appointment depends on the possession of a rabbinical certificate, and on Shabbat or other holy days no killing can take place.

Not always are the dietary laws clear and explicit and there is often room for interpretation, especially with regard to insects as food. Popularly considered 'trefah' (unfit for human consumption) locusts and scale insects are an exception and some Jewish scholars firmly believe that in the passage [33] "examine beast, fowl, locusts, and fish to determine whether they are permitted...", the term locusts stands for insects generally, while others apply it to just four species of locusts. Jewish dietary laws apply to everyone in the community, so that no exceptions for children, women or old folk are permitted, as long as a human life is not endangered. The protection of human life, however, overrides all dietary discipline and for priests and dealings with priests additional dietary rules apply.

Christian 'Seventh Day Adventists' have adopted many of the Jewish/Biblical dietary laws, but while to the Jew there is a place for wine, coffee, and tea (at least for those old enough to have been given complete religious responsibility), Seventh Day Adventists declare all intoxicating and addictive drinks prohibited. Gluttony and drunkenness are, of course, also forbidden to Jews.

## Discussion

### General remarks

Different workers have different opinions on what constitutes a "food taboo". Generally speaking, a taboo prohibits someone from doing something, e.g., "touching a sacred person, killing a certain animal, eating certain food, eating at certain times" [39]. Taboos represent "unwritten social rules that regulate human behaviour" [14] and define the "in-group" [20]. According to Barfield [40] there may be as many as 300 reasons for particular avoidances (amongst them not wanting to look like a food item, special place of food item in myth or history, food item perceived as dirty, predatory, humanlike etc.), which can magnify effects of seasonal or other restrictions on nutritional intake and may put women at nutritional risk during critical periods in their reproductive cycle.

If the avoidance of a certain food item provides the food avoider with an immediate result, for instance absence of an allergic reaction, we can assign a proximate cause to the food item in question. However, if the consequences of a food taboo are not immediately visible and may take months, years, or even generations to manifest themselves, we have to speak of ultimate causes. For researchers of food taboos, the often unsurmountable difficulty is that proximate and ultimate causes of food taboos may overlap [17] and, in fact, cannot always be separated.

It can be seen from these remarks that a discussion of food taboos is possible in a variety of ways with a variety of foci. By using the examples given above in this paper, the author wishes to highlight certain reasons, which seem to have been involved in the establishment of food taboos in those cultures examined (but may have been at the root of food taboos in other cultures as well). Discussing the examples in this way, a kind of classification results that might well be generally applicable to societies (not part of this investigation), in which food taboos exist.

### Food taboos for certain members of the society and to highlight special events

Any interpretation of food taboos has to consider the region they operate in, the era or circumstances they came into existence, or, in other words, the food history of a people [7,8,41,42]. Desert locusts, having been common and sustained ancient Israelites in a dry land, are not taboo, but why should other insects be taboo? Rational explanations are not always possible and what to one group is strictly taboo, to another may be perfectly acceptable [43]. Some food taboos evolved in connection with attempts to steer or control man's destiny [44] and attempts to put some "order" into the occurrence of and reason(s) behind food taboos must realize that food taboo categories are not clear-cut. Food taboos, based on religious beliefs for example, may have a health-related root and taboos restricting certain foods to men may be an expression of male dominance or differences in skills between the sexes.

Taking a look at the ubiquity of food taboos, we notice that sometimes taboos affect all sections of the population at all times: Jewish dietary laws [45] and the basic Hindu regulation of "no meat, no fish, no eggs" are cases in point. Occasionally, ubiquitous food taboos become suspended or are enforced periodically as with the Friday for the Catholic Christians, when no meat but fish only is to be consumed and the pre-Easter weeks of lent, when meat of warm-blooded animals should not be eaten. The annual Yom Kippur with its total ban of food and liquid intake as a periodical food taboo event (cf., definition of the word taboo [39]) also comes to mind, but this total stop of food and liquid intake is a special case.

Frequently, food taboos affect males or females, leaders or subjects, children or widows and widowers differently; in other words they are distributed unevenly. Food taboos, as we have seen in the examples of the Orang Asli in Malaysia [24], mid-west state Nigerians [30], or parts of the Congo [46], may change throughout a person's lifetime with age in a predictable manner, as accepted and expected by society.

Food taboos frequently accompany 'coming-of-age' or initiation ceremonies [47]; they can also be prescribed at times of drought, flooding or lunar and solar eclipses, and many more events. Thus, one of the aims of food taboos is to highlight particular happenings, making them memorable. In fact, the vast majority of all food taboos come under this group of "specific events" and one of its various sub-categories. Food taboos at menstruation, during and after pregnancies, on the sickbed in times of illness, in times of mourning, in preparation for a wedding, or before combat are commonly encountered [48]. Persons of Asian descent traditionally perceive health in connection with the bodily balance of 'hot and cold' and, thus, when under the influence of disease or pregnancy, would avoid food items considered 'hot', which may even include iron tablets [49].

### Food taboos to protect human health

When a particular taboo is regarded as God-given, as a form of instruction or command from the "Supreme" and thus play a role in the cultural or religious belief system [14], then it is usually seen as part of a 'package' to protect the believers, to safeguard them against evil [20-23]. To doubt, even to ask any questions about the reasons behind the taboo is seen as blasphemous. Likewise, in tribes with totem beliefs, it follows that it has to be taboo to eat the totem animal, as otherwise it could take revenge and adversely affect the whole tribe [42]. However, irrespective of the God-given rules or advice, people must have noticed changes in the behaviour of persons that consumed certain food items. Such behavioural and/or emotional consequences of certain foods must have been recognizable not only to the consumer of the food, but also to her/his company and could have been the origin of such seemingly God-given guidelines. For instance, food items involved in IgE-mediated allergies (like, for instance, shrimp: [50]) should have been easily identifiable and then could first have led to their avoidance and, secondly, to a total ban of them.

Eating to regulate emotions has been listed as one of the five classes of "emotion-induced changes of eating" by Macht [51] and IgE-mediated atopic diseases are known to be associated with depression [52] and suicide rate [53]. An increase of unsaturated fatty acids in the diet has been found to be correlated with decreased violent behaviour [54] and an exposure to sunflower seeds [55] and colorants derived from the fungus *Monascus ruber *[56] can cause asthma attacks. Finally, low glycaemic meals have been reported to improve memory and ability to sustain attention [57], features that might not have gone unnoticed by our forebears in earlier times and could have led to the avoidance or recommendation not to consume certain food items.

As scientists we are obliged to probe, to scrutinize, to question and although many food taboos do not appear to have a health-related, 'rational' explanation, some clearly have become established, because of the aim to protect the health of an individual (and this would equally apply to Modena's recently suggested "anti-taboo" concept in choosing food denominations: [58]). Taboos of the Hindu related to collecting fruits and breaking plants after sunset go back to times when no artificial lighting was available and, therefore, it must have been outright dangerous to pick fruits at night. Consequently, it would make perfect sense to taboo the collecting of fruit after dark. Nausea, vomiting, diarrhoea, cramps, and maybe death, whether rightly or wrongly, were frequently considered to be some of the after-effects of ingesting certain foods [41].

In some cases the threat to a person's health may be obvious and demonstrable with modern medical, chemical, and other analytical techniques, but of course it was not always like this. Amazon and coastal fishermen, for example, declare mostly carnivorous, especially piscivorous, fishes taboo: we know now that their place high in the food pyramid renders them particular rich in contaminants and toxins [17]. Alcohol, another example, is an addictive poison and as such is taboo for children of most societies. Snakes and other venomous or dangerous creatures had better be left alone as the risk of procuring them for food can outweigh their nutritional value. A utilitarian reason to despise swine, as it competes with humans for food and water in dry lands, has been put forward by Harris [7], but pork is taboo to many people, because pigs tend to harbour masses of sickness-causing parasites. Moreover, it is claimed that pig meat contains substances, which have been linked to high blood pressure, atherosclerosis, rheumatism, arthritis, boils, asthma and eczema. Apparently, soldiers fighting in North Africa during World War II began to increasingly suffer from toxic ulcera of the legs as long as there was pork in their diet. When their food was pork-free, the ulcera disappeared [59].

### Food taboos during pregnancy and food changes over the course of the menstrual cycle

Declaring certain foods taboo because they are thought to make a person sick, is also the basis for the many food taboos affecting pregnant women. Largely linked with the realms of mind and 'psyche', the taboos of not eating cryptic fish amongst the Trobriand Islanders or watermelon and other fruits amongst the Onabasulu are actually meant to protect the health of the pregnant woman and her offspring and thought to ease the process of birth-giving, even if modern nutritionists completely disagree. Likewise, the rule of the Orang Asli that young people can only cope with small animals like snails, mice and rats as food, because their spirits are also small and for that reason are not likely to do much harm to a small child's spirit, is designed to protect human life.

Yet, it is often pregnant and lactating women in various parts of the world that are forced to abstain from especially nutritious and beneficial foods (Mexico: [60]; Indonesia: [61]; Korea: Lee H.-I., pers. comm.). Although it is not clear why and how exactly these restrictions came to be accepted (see below), pregnant women do not always adhere to them. Amongst the Lese-women of the Ituri forest of Africa, women cope with these restrictions by either secretly discounting them or by eating prophylactic plants that supposedly prevent the consequences of eating the tabooed foods [62]. Flaunting taboos has also been reported by Alvard [63], who then suggested that food taboos would be of little value to nature conservation (but see the evidence to the contrary by Colding and Folke [14]).

The fact that women throughout the world (with few exceptions) display a slightly but significantly reduced calorific intake around the time of ovulation has been noted for a long time and formed the topic of a recent review by Fessler [64]. He used the term "periovulatory nadir" for the phenomenon and concluded that it was linked to increased locomotor activity, interest in wanderlust, "a desire to meet new people (particularly men)". Regrettably, it is not known if specific food items are being avoided, perhaps even subconsciously, at the time of the periovulatory nadir.

### Food taboos as an ecological necessity to protect the resource

As hinted upon earlier and demonstrated in several studies, most notably [14-19], food taboos frequently seem to have an ecological background, which according to Harris [7] is based on utilitarian principles. On the one hand, they may lead to a fuller utilization of a resource and on the other they can lead to its protection. If North West American Inuit and Nootka Indians both hunt and eat the whale, it makes good ecological sense when the Tlingit Indians of the same region regard the giant sea mammal as taboo and look for food on land [65]. Some ecological consequence can also be ascribed to the custom amongst the Ka'aor Indians of the northern Maranhao (Brazil) of allowing only menstruating women, pubescent girls, and parents of newborns to consume the meat of tortoises [66] and the fact that amongst the indigenous people of Ratanakiri (Cambodia) different food taboos operate even between neighbouring villages [67]. Of 70 existing examples of species-specific taboos, identified and analysed by Colding and Folke [14], 30% were found to prohibit the use of species listed as threatened by the IUCN Red Data Book.

In the same vein, if women and children, as in the Orang Asli, eat only small animals while older people also consume bigger species, a measure like this would distribute ecological pressure more evenly across a greater number of consumable species. This can lead to a situation, in which females are only permitted plants and insects as food, while the menfolk are free to ingest meat, egg, and fish [7]. The regulations amongst the Canadian Netsilik [68] that sea-mammal and terrestrial mammal must never be eaten on the same day and amongst Jews that milk and milk-containing foods cannot be consumed together with meat, have an ecological ring. Clearly, sustainability of a resource is served by the taboo not to eat the young and its parent and by the Hindu custom of not totally finishing a plate, so that there is always some plant material left over for Nature (e.g., seeds). To safeguard a resource for a time of crisis may be the reason, why certain fishes of the Amazon are not normally eaten, but spared [69].

### Food taboos in order to monopolize a resource

Declaring a food item taboo for one section of the population, can of course, lead to a monopoly of the food in question by the remainder of the population [7]. For purely egoistic reasons men may declare meat and other, to them, delicacies taboo "for others". That this is the main reason for some food taboos affecting mainly women and children, is suspected by [30]. Traditional healers in Nigeria sometimes attribute childhood ailments to breaking the food norms [70] and in Senegal women and children, but not men, must avoid poultry products. That this can lead to a shortage of adequate supplies of essential nutrients especially in the most vulnerable group of the rural population is self-understood [71].

The fact that in many societies alcohol-drinking women are poorly respected (while for men alcohol consumption is regarded as normal), in essence, seems little different from the Australian aboriginal practice that native honey (a rare and sweet delicacy) is seen as something fit only for the old and wise men. Amongst the Bolivian Siriono, there are "hundreds of food taboos", but they apply only very loosely to the elderly, who can break the taboos. This ensures their welfare and survival when no longer able to hunt for the 'right food' [72].

### Food taboos as an expression of empathy

Empathy, i.e., feeling for and with the poor animal that is to have its life terminated for the selfish reason of devouring it, is yet another powerful reason for certain food taboos to have come into existence. In many societies, pet animals enjoy a greater degree of protection and are more likely to be given "taboo" status than individuals that are unfamiliar and "unrelated". It is almost as if "humanness" rubs off and the pet becomes regarded as an "honorary human".

Hindu religious thought with its belief of re-incarnation even goes a step further and basically does not distinguish between human and animal with regard to their souls – only the packaging is seen to differ. It follows that by eating an animal, a Hindu could indeed, to put it bluntly, be eating a deceased relative. And that -with few exceptions where endocannibalism was the accepted practice and parts of a human corpse were ritually consumed as in certain tribes of Papua Niugini- is almost everywhere a taboo [73-75].

### Food taboos as a factor in group-cohesion and group-identity

Finally, it ought to be mentioned that any food taboo, acknowledged by a particular group of people as part of its ways, aids in the cohesion of this group, helps that group stand out amongst others, assists that group to maintain its identity and creates a feeling of "belonging". Thus, food taboos can strengthen the confidence of a group by functioning as a demonstration of the uniqueness of the group in the face of others.

Food taboos and food habits can persist for a very long time and can be (and have been) made use of in identifying cultural and historical relationships between human populations [76,77]. It has, for instance, been suggested that the food taboos of both Jews and Hindus reflect not the nutritional needs, but the explicit concerns of the pastoral peoples' that they once were [78].

## Conclusion

In our increasingly international world, it is essential that we know and understand food taboos of societies other than and in addition to our own. In a world, in which many persons still go hungry, it is important to realize that numerous societies impose restrictions on what is acceptable as food and that in most cases the full food potential of a given environment is not being made use of. Food restrictions can affect the nutritional status of a community or a subsection within it. There may be sound reasons for prohibiting certain food items as we have demonstrated in this paper, but declaring some food items taboo can equally well be a form of suppression by a more dominant sector of the society. To explore the operating food taboos from historic, hygienic, and social perspectives must be the aim of any study that deals with the problem of community food culture [10,14,79,80]. In the words of Drewnowski and Levine [80]: "There is a need for further discussions of the economics of food choice".

## Competing interests

The author declares that he has no competing interests.

## Author's contributions

The single author of this paper (VBM-R) is responsible for every aspect of the research, the conclusions, and the writing of the paper.
